# Tailoring lipid nanoparticles for T-cell targeting in allergic asthma: Insights into efficacy and specificity

**DOI:** 10.1016/j.ejpb.2024.114242

**Published:** 2024-03-03

**Authors:** David C. Jürgens, Joschka T. Müller, Anny Nguyen, Olivia M. Merkel

**Affiliations:** a Department of Pharmacy, Ludwig-Maximilians-University Munich, Butenandtstrasse 5-13, Haus B, 81377 Munich, Germany; b Center for NanoScience (CeNS), Ludwig-Maximilians-University Munich, 80799 Munich, Germany; c Ludwig-Maximilians-University Munich, Member of the German Center for Lung Research (DZL), Germany

## Abstract

Asthma impacts over 300 million patients globally, with significant health implications, especially in cases of its allergic subtype. The disease is characterized by a complex interplay of airway inflammation and immune responses, often mediated by Th_2_ cell-related cytokines. In this study, we engineered lipid nanoparticles (LNPs) to specifically deliver therapeutic siRNA via the transferrin receptor to T cells. Strain-promoted azide-alkyne cycloaddition (SPAAC) was employed for the conjugation of transferrin ligands to PEGylated lipids in the LNPs, with the goal of enhancing cellular uptake and gene knockdown. The obtained LNPs exhibited characteristics that make them suitable for pulmonary delivery. Using methods such as nanoparticle tracking analysis (NTA) and enzyme-linked immunosorbent assay (ELISA), we determined the average number of transferrin molecules bound to individual LNPs. Additionally, we found that cellular uptake was ligand-dependent, achieving a GATA3 knockdown of more than 50% in relevant *in vitro* and *ex vivo* models. Notably, our findings highlight the limitations inherent to modifying the surface of LNPs, particularly with regard to their targeting capabilities. This work paves the way for future research aimed at optimizing targeted LNPs for the treatment of immunologic diseases such as allergic asthma.

## 1 Introduction

Globally, more than 300 million patients are diagnosed with asthma, a condition whose prevalence is on the rise and accounts for a minimum of 250,000 deaths each year. [1,2] Common symptoms include reversible airflow obstruction, bronchial hyperresponsiveness, and airway inflammation. [3] The primary subtype, known as Type 2 asthma, can be further classified into Th_2_-high and Th_2_-low subtypes based on the concentration of Th_2_ cell-related cytokines. Immunomodulating cytokines including Interleukin (IL) 4, 5, and 13 often lead to elevated levels of eosinophils and Immunoglobulin E (IgE). [4] As a consequence, sensitivity to allergens is enhanced, epithelial cells are stimulated, and immune response cells such as mast cells, eosinophils, and basophils are attracted. These effects also bring about structural changes in the epithelium and the underlying matrix, resulting in symptoms such as shortness of breath, wheezing, coughing, and sputum production. [4]. Mechanistic studies have revealed that expression of the transcription factor GATA Binding Protein 3 (GATA3) controls the expression of IL4, IL5, and IL13 in CD4^+^ Th_2_ cells. [5] In the context of asthma, Nakamura et al. have demonstrated that increased GATA3 expression can be measured in patients with significant colocalization in T cells (approximately 60 % to 90 %). [6] Although the role of other cell types in GATA3 expression remains a subject of ongoing debate, the targeting of GATA3 using DNAzyme technology as a potential asthma treatment has successfully advanced through Phase 2b clinical trials. [7] With this understanding, and acknowledging the pivotal role of GATA3 in CD4 + Th_2_ cells, our research is directed towards the efficient delivery of small interfering RNA (siRNA) specifically into these cells. Our aim is to inhibit the synthesis of Type 2 cytokines, a strategy whose efficacy has been demonstrated *in vitro, ex vivo*, and *in vivo* using polymer-based carriers. [8–11].

Lipid Nanoparticles (LNPs) serve as promising drug delivery systems for various RNA types, including siRNA. [12] We developed a targeted LNP formulation, inspired by the clinically-approved Onpattro® LNP composition. Acknowledging Onpattro’s proven ability to facilitate endosomal escape—a known bottleneck in drug delivery—we retained three of its original four lipid components: D-lin-MC3, Cholesterol, and DSPC. [13] The forth component, the PEGylated lipid was modified as lined out below.

The transfection of T-cells, however, proves challenging. [8,10,11,14] Therefore, we modified the concentrations of polyethylene glycol (PEG)ylated lipids in LNPs and introduced a targeting ligand to enable active targeting of the LNPs to Th_2_ cells, with the aim of enhancing cellular uptake and efficacy. [15] Transferrin (Tf) was chosen as the targeting ligand due to the abundant expression of transferrin receptors (TfR) on the surface of Th_2_ cells particularly during T-cell activation. [16] Transferrin’s targeting ability has previously been demonstrated in a clinical phase 1 study by Johnson et al., which shows its efficacy in solid tumor therapy. [17] For our study we employed the strain-promoted azide-alkyne cycloaddition (SPAAC) reaction for conjugation and opted for PEGylated lipids as anchors for conjugation. These function as spacers between the LNP and the targeting ligand, aiming to enhance cell interaction and specificity while reducing protein corona formation, as reported for Trastuzumab HSA-PEG conjugates. [18] We also employed post-insertion functionalization to target the LNPs, thereby rendering the underlying LNP framework adaptable for ligand exchanges. Finally, we utilized Jurkat cells as immortalized human lymphocyte cell line to validate successful transferrin-dependent cellular uptake and downregulation of the transcription factor GATA3.

## 2 Material and methods

### 2.1 Preparation of siRNA-loaded LNPs

The formulation of siRNA LNPs was chosen based on the lipid composition of the clinically approved Onpattro® formulation. The following lipids were used: ionizable lipid (6Z,9Z,28Z,31Z)-heptatriaconta-6,9,28,31-tetraen-19-yl 4-(dimethylamino)butanoate (Dlin-MC3-DMA, MedChemExpress, Monmouth Junction, USA), helper lipid 1,2-distearoyl-*sn*-glycero-3phosphocholine (DSPC, Sigma Aldrich, Taufkirchen, Germany), cholesterol (Corden Pharma, Taufkirchen, Germany), and the PEGylated lipid 1,2-distearoyl-*sn*-glycero-3-phosphoethanolamine-N-[azido(polyethyleneglycol)-2000] (ammonium salt) (DSPE-PEG 2000 Azide, Sigma Aldrich, Taufkirchen, Germany).

The lipids were dissolved in ethanol to achieve a final concentration of 2 mM. Subsequently, the lipids were combined in the following molar ratios: 50:10:39.5:0.5 mol%, 50:10:38.5:1.5 mol%, and 50:10:37.5:2.5 mol% for Dlin-MC3-DMA, DSPC, cholesterol, and DSPE-PEG 2000 Azide, respectively. The siRNA used in the study was dissolved in 25 mM sodium acetate buffer (pH 4) to achieve a nitrogen-to-phosphate (N/P) ratio of 3 for siRNA. The N/P ratio represents the ratio of the amine groups present within the ionizable lipid to the phosphate groups in the siRNA backbone. The LNP formulation process involved the assembly of lipid components and siRNA. The siRNA LNPs were manufactured using a Staggered Herringbone mixing microfluidic chip (Fluidic 187, Microfluidic ChipShop, Jena, Germany). The mixing process was carried out at a total flow rate (TFR) of 3 mL/min and a flow rate ratio (FRR) of 3:1 (v/v) for the aqueous and organic phases, respectively. After microfluidic mixing, the resultant LNPs were subjected to dialysis using a 3.5 kDa molecular weight cutoff Pur-A-Lyzer™ Maxi Dialysis kit (Sigma Aldrich, Taufkirchen, Germany). Dialysis was performed over night against RNase-free phosphate-buffered saline (PBS) with a pH of 7.4 and maintained at 4 °C. The final LNP suspension was then sterile filtered using a 0.22 µm Acrodisc® syringe filter (Pall, Dreieich, Germany). Following the dialysis and filtration steps, the LNP samples were stored at 4 °C until further experimental use. An increase in volume was observed after dialysis. The volume increase factor was quantified for each LNP sample and utilized for subsequent analyses of LNP recovery and encapsulation efficiency.

### 2.2 Transferrin conjugation and purification

Human transferrin (Sigma Aldrich, Taufkirchen, Germany) was dissolved at a concentration of 10 mg/mL in PBS. Subsequently, a 5-fold molar excess of a 10 mM dibenzocyclooctin-*N*hydrox-ysuccinimidylester (DBCO-NHS, Sigma Aldrich, Taufkirchen, Germany) in anhydrous dimethyl sulfoxide (DMSO, Sigma Aldrich, Taufkirchen, Germany) was added and the solution was stirred for 2 h at room temperature. Purification of the obtained Tf-DBCO solution was performed using 10,000 MWCO centrifugal filters (Cytiva Sweden AB, Uppsala, Sweden) against PBS. Quantification of DBCO incorporation was executed spectrophotometrically (NanoDrop One, Thermo Fisher Scientific, Darmstadt, Germany) through assessment of the relative absorbance A280/A309. A complete absorption-spectrum was recorded of the resulting Tf-DBCO solution based on following formula:

$$\frac{DBCO}{\;Transferrin\;}=\frac{A309x\epsilon_{Transferrin\;}}{(A280-CFx309)\times \epsilon_{DBCO}}$$

∈*_Transferrin_*: the molar extinction coefficients of Transferrin at 280 nm: 112,000 *M*^−1^ *cm*^−1^

*∈_Dbco_:* the molar extinction coefficients of DBCO at 309 nm: 12,000 *M*^−1^ *cm*^−1^

A280: the absorption value sample at 280 nm

A309: the absorption value sample at 309 nm

CF: the correction factor of DCO at 280 nm; 1.089

The purity of Tf-DBCO was confirmed by measuring the absorbance at 309 nm in the flow through solution after the spin filtration process. Subsequently, LNPs were stirred for 4 h with a molar excess of Tf-DBCO solution. The quantity of added Tf-DBCO remained consistent across different LNP formulations, thereby conferring the azide-containing moiety the role of reaction-determining functional group. The molar ratio of DBCO to azide was maintained at a 4-fold molar excess of DBCO, with calculations based on the 1.5 % molar LNP formulation. After conjugation, the excess transferrin-DBCO was removed by centrifugation (1000 rcf, 15 min, 21 °C) applying spin columns (Vivaspin 6, Sigma Aldrich, Taufkirchen, Germany) with a 100 kDa molecular weight cut-off. The LNPs were washed 4 times with PBS. The flow-through of each purification step and the purified LNPs were kept at 4 °C until analysis for free Tf-DBCO by transferrin ELISA. The transferrin ELISA (ab288174, Abcam, Berlin, Germany) was performed according to manufacturer’s instructions applying a sample dilution of 1:10,000. Measurements were performed in technical duplicates. Values are given as mean values ± standard deviation (SD).

### 2.3 Hydrodynamic diameters and zeta potentials of siRNA loaded LNPs

The hydrodynamic diameter and polydispersity index (PDI) of the LNPs were determined through dynamic light scattering (DLS) using a Zetasizer Advance Ultra (Malvern Instruments, Malvern, UK) equipped with backscatter angle detection using disposable cuvettes. LNP samples prepared with scrambled siRNA (Integrated DNA Technologies, Coralville, USA). Triplicate measurements (n = 3) were conducted, and the data analysis was carried out using ZS Xplorer software (v. 3.20). The results are reported as the mean size (nm) ± SD.

Nanoparticle tracking analysis (NTA) measurements were utilized to assess the particle count of the LNPs. Samples were diluted 1:10 for both azide LNPs and transferrin LNPs and introduced into the NanoSight NS300 device (Malvern Instruments, Malvern, UK) using the syringe pump. The particle concentration is expressed as particles/mL ± SD (n = 3). The zeta potential of the LNPs was evaluated employing Phase Analysis Light Scattering (PALS) with the Zetasizer Advance Ultra (Malvern Instruments, Malvern, UK). Each formulation’s 100 µL sample was diluted 1:7 with PBS and transferred to a non-disposable capillary cell from Malvern Instruments. ZS Xplorer software (v. 3.20) facilitated the data analysis, yielding the average charge (mV) ± SD. The measurements were performed in triplicate, each comprising 30 runs for every sample (n = 3).

### 2.4 Encapsulation efficiency

To determine the encapsulation efficiency and RNA content in LNPs, the RiboGreen™ assay was employed. In brief, a volume of 5 µL from each LNP sample was transferred into a black 348-well plate and subsequently adjusted to a final volume of 10 µL using either TE buffer (Sigma Aldrich, Taufkirchen, Germany) at pH 7.5 for encapsulation efficiency evaluation, or a solution of 2 % Triton X-100 (Sigma Aldrich, Taufkirchen, Germany) in TE buffer for determination of total RNA content. Standard curves were prepared in both buffers using a serial dilution of 10 µg/mL siRNA solution. The plate was then placed in a shaking incubator at 37 °C for 60 min. Following the incubation, 10 µL of the fluorescent dye Quant-iT™ RiboGreen™ (Invitrogen™, Thermo Fisher Scientific, Schwerte, Germany), diluted at a ratio of 1:100, was added to each well. After a 10-minute incubation period, the fluorescence intensity was measured using a microplate reader (Tecan Spark, TECAN, Männedorf, Switzerland) at an excitation wavelength of 480 nm and an emission wavelength of 525 nm. The results are expressed as a percentage ± SD. Technical triplicates were utilized for the measurements (N = 3).

### 2.5 Cell culture

For transfection experiments, the human T lymphocyte cell line Jurkat was used, which were generously provided by Prof. Heissmeyer from the Institute for Immunology, Biomedical Center Munich. The cells were cultured using RPMI media (Sigma Aldrich, Taufkirchen, Germany) supplemented with 10 mM HEPES, 1 mM sodium pyruvate, heat-inactivated fetal bovine serum (FBS) at a concentration of 10 % (Thermo Fisher Scientific, Darmstadt, Germany), 4.5 g/L glucose, and Penicillin-Streptomycin at a concentration of 1 % (Thermo Fisher Scientific, Darmstadt, Germany) in phosphate-buffered saline (PBS) obtained from Thermo Fisher Scientific, Darmstadt, Germany. Jurkat cells were routinely subcultured and maintained in a controlled incubator environment set to humidified air with 5 % CO_2_ at a temperature of 37 °C. Subculturing was performed every 2–3 days to sustain a cell concentration within the range of 0.5 x 10^6^ to 1.5 x 10^6^ cells/mL, ensuring optimal conditions for subsequent experiments.

### 2.6 Cellular uptake

For the cellular uptake assay, a total of 250,000 Jurkat cells in 500 µL of growth medium were seeded per well in a 48-well tissue plate. 24 h after seeding cells were subjected to transfection. The LNPs utilized for this study were formulated with a composition consisting of 30 % AF647-labeled siRNA (Integrated DNA Technologies, Coralville, USA) and 70 % scrambled siRNA. The transfection process involved the use of both transferrin-conjugated and non-conjugated LNPs. The LNPs were diluted in sterile PBS to achieve a final siRNA dose of 100 pmol in 100 µL of PBS pH 7.4, based on the encapsulated RNA content. For blank samples, 100 µL of sterile PBS was added to each well. As positive controls, Lipofectamine 2000 (Thermo Fisher Scientific, Darmstadt, Germany) was employed in accordance with the manufacturer’s instructions, considering the siRNA LNP doses used. In the context of a receptor competition assay, 1 mg/mL free transferrin was added to each respective LNP sample.

Following a 2 h incubation period in a controlled environment of humidified air with 5 % CO_2_ at 37 °C, the cell suspensions were transferred to 1.5 mL Eppendorf tubes and centrifuged (400 rcf, 5 min, 21 °C). Following centrifugation, the supernatant was aspirated, and the cells were washed twice using 500 µL of PBS (400 rcf, 5 min, 21 °C). Subsequently, the cells were resuspended in 400 µL of PBS containing 2 mmol of EDTA (Sigma Aldrich, Taufkirchen, Germany) and subjected to another round of centrifugation (400 rcf, 5 min, 21 °C). Flow cytometry analysis was performed utilizing an Attune Nxt instrument (Thermo Fisher Scientific, Darmstadt, Germany), with data acquisition from 50,000 cells using a 643 nm excitation laser and the RL1 filter. The results of the flow cytometry analysis are presented as the average mean fluorescence intensity (MFI) of three technical replicates (n = 3) derived from three replicate measurements (N = 3), each with its corresponding SD.

### 2.7 Cellular distribution

To visualize cellular distribution, 125,000 Jurkat cells were seeded in a 48-well tissue culture plate with 500 µL of growth medium and immediately subjected to transfection. LNPs used in this study consisted of a blend of 30 % AF647-labeled siRNA and 70 % scrambled siRNA formulated into LNPs with a PEGylated lipid content of 1.5 %. For transfection, a total of 50 pmol of siRNA, employing both transferrin-conjugated and non-conjugated LNPs, was used. To each well, 100 μL of LNPs were added and incubated for 24 h. One hour prior to the readout, 75 nM LysoTracker Yellow (Thermo Fisher Scientific, Waltham, USA) was added for endolysosomal staining. For sample processing, cells were transferred onto a microscope glass slide using a Cytospin 3 centrifuge (Marshall Scientific, Hampton, USA) at 600 rpm for 5 min. Afterward, the medium was aspirated, and the cells were washed three times with PBS, then fixed with 4 % paraformaldehyde (PFA, 37 %, Sigma-Aldrich, Taufkirchen, Germany) for 10 min at room temperature. Residual PFA was removed by washing three times with PBS. After fixation, nuclei were stained with 0.5 μg/mL 4′,6-Diamidin-2-phenylindol (DAPI, Thermo Fisher Scientific, Waltham, USA) in PBS for 10 min in the dark. Subsequently, cells were washed three times with PBS and mounted onto a coverslip for confocal microscopy analysis using FluorSafe (Sigma-Aldrich, Taufkirchen, Germany) and visualized with a Leica SP8 confocal microscope (Leica, Wetzlar, Germany). Analysis was conducted using the Leica Application Suite X.

### 2.8 GATA3 knockdown in Jurkat cells

For the GATA3 gene silencing assay, a total of 250,000 Jurkat cells were seeded in 500 µL of growth medium per well in a 48-well plate. Upon seeding, the cells were immediately subjected to transfection. The LNPs utilized for this study were formulated with either an equally blended composition of GATA3 siRNA sequences Hs_GATA3_8 and Hs_GATA3_9 (Qiagen, Hilden, Germany) or scrambled siRNA (Integrated DNA Technologies, Coralville, USA). The transfection process involved the use of both transferrin-conjugated and non-conjugated LNPs. The LNPs were diluted in sterile PBS to achieve a final siRNA dose of 100 pmol in 100 µL of PBS, based on the encapsulated RNA content. 24 h after transfection the total RNA was isolated using RNeasy mini kits (Qiagen, Hilden, Germany) according to the manufacturer’s instructions. The cDNA was synthesized from total RNA with a high-capacity cDNA synthesis kit (Applied Biosystems, Waltham, Massachusetts, USA) and was then diluted 1:10 for qPCR analysis. The RT-qPCR (QuantStudio 3 Real-Time PCR, Thermo Fisher Scientific, Waltham, Massachusetts, USA) was performed using SYBR™ Green PCR master mix (Thermo Fisher Scientific) with primers for human GATA3 (Biorad, Hilden, Germany) and human β-actin (Qiagen, Hilden, Germany). For normalization the ΔΔCt method was applied. Amplification and data analysis was performed using a QuantStudio 3 Real-Time PCR (Thermo Fisher Scientific, Waltham, Massachusetts, USA). Cycle thresholds were acquired by auto-setting with the qPCR software (Thermo Fisher Scientific, Waltham, Massachusetts, USA). Values are given as mean values ± SD of biological and technical triplicates (n = 3, N = 3).

### 2.9 GATA3 knockdown in primary CD4^+^ Th_2_ cells

Peripheral blood mononuclear cells (PBMCs) were isolated from freshly obtained buffy coat samples (DRK, Berlin, Germany) via ficoll-paque gradient centrifugation (Ficoll® Paque Plus, GE Healthcare). Subsequently, naïve CD4 + T cells were isolated using magnetic bead separation with the EasySep™ Human Naïve CD4 + T Cell Isolation Kit II (STEMCELL Technologies, Saint Égréve, France), following the manufacturer’s instructions. The isolated naïve CD4 + T cells (1 x 10^6^ cells/mL) were cultured in ImmunoCult™-XF T Cell Expansion Medium (STEMCELL Technologies, Saint Égréve, France) and stimulated with anti-CD3/CD28 Dynabeads™ (Human T-Activator CD3/CD28, Thermo Fisher Scientific Waltham, Massachusetts, USA). To induce Th_2_ differentiation, the cell culture medium was supplemented with rIL-4 and anti-IFN-γ (ImmunoCult™ Human Th_2_ Differentiation, STEMCELL Technologies, Saint Égréve, France). Cell density was maintained at 1 x 10^6^ cells/mL by adjusting with freshly supplemented medium every 3–4 days, with restimulation performed every 7 days.

For GATA-3 knockdown, 500,000 Th_2_ cells were seeded per well in a 48-well tissue culture plate with 500 µL of growth medium. Activation was achieved using Dynabeads™ Human T-Activator CD3/CD28 (Thermo Fisher Scientific, Waltham, Massachusetts, USA) at a 1:1 bead-to-cell ratio, in conjunction with 30 U/mL rIL-2 (Human IL-2 research grade, Miltenyi, Berlin, Germany), adhering to the manufacturer’s protocol. After 24 h of activation in a humidified atmosphere with 5 % CO_2_ at 37 °C, cells were transfected with 1.5 % PEGylated lipid LNP compositions. The transfection, RNA isolation, cDNA synthesis, and RT-qPCR procedures were carried out similarly to those for Jurkat cells, and pooling cell lysates prior to cDNA synthesis. Results are presented as mean values ± SD of biological and technical triplicates.

#### Statistical Analysis

All experiments were performed in triplicates. All results are presented as mean value ± SD. One-way ANOVA with Tukey posthoc post-test was conducted in GraphPad Prism due to equal sample sizes (GraphPad Software, La Jolla, USA) to calculate p-values with 95 % confidence.

## 3 Results and discussion

### 3.1 LNP and Tf-LNP preparation

We initiated our study by varying the concentration of DSPE-PEG azide in the LNP formulation, starting at 1.5 %. We then incrementally increased and decreased this concentration by 1 % and adjusted the cholesterol concentration in the formulation accordingly (Table S1). After formulating the LNPs, we introduced a targeting ligand to the carrier system to enhance both transfection efficacy and specificity, thereby reducing potential adverse effects and off-target consequences induced by the formulation. [15] For LNP modification with a targeting ligand, various strategies are described in the literature. Okamoto and colleagues formulated LNPs and introduced Fragment Antigen Binding fragments (Fab’) of anti-Heparin-Binding EGF-like Growth Factor (anti-HB-EGF) antibody by incubation and mixing of crude siRNA LNPs with DSPE-PEG Fab’ conjugates for 16 h. [19] Li and colleagues adopted a different strategy for transferrin receptor targeting. They used a lipid composition with 2 % Tf-Cholesterol-PEG conjugate for LNP formulation from the very beginning. [20].

For our study the azido group of the PEGylated DSPE was exploited for click chemistry-driven conjugation with transferrin following the LNP manufacturing, as illustrated in Figure S1. The purity of Tf-DBCO was assessed spectrophotometrically by examining the remaining DBCO in the flow through after centrifugation. After the third flow through did not show a measurable concentration of free DBCO, as illustrated in Table S2, the solution was used to conjugate Tf to the azide-modified LNPs. The evaluation of DBCO-Tf revealed an average of 6.5 DBCO molecules per transferrin molecule. The employed SPAAC is advantageous due to the absence of Cu(I) as a catalyst, making the approach more suitable for potential *in vivo* application with lower toxicity. [21] The advantages of post-manufacturing modification were demonstrated by Peer and colleagues, who utilized this strategy for successful targeting of specific leukocyte subsets. [22] This method aims to prevent potential degradation of transferrin due to ethanol or the acidic nature of the aqueous buffer and to bypass any unpredictable impacts of the DSPE-PEG transferrin conjugate on LNP formation during microfluidic assembly. Additionally, we strive to preserve the availability of the transferrin molecule on the LNP surface to effectively target the transferrin receptor, a receptor known to be expressed on the surface of Th_2_ cells. [11].

As shown in Table 1 the measured transferrin concentration increased depending on the used PEGylated lipid. Hence, mean concentrations of 1.16 µg/mL, 1.88 µg/mL, and 7.30 µg/mL were measured for the 0.5 %, 1.5 %, and 2.5 % DSPE-PEG azide formulations, respectively. To confirm the removal of unbound transferrin and to determine the quantity of conjugated transferrin on the LNP surface, we employed a transferrin ELISA (Figure S3). To further explore the potential influence of DSPE-PEG azide concentration on the number of transferrin molecules present on the LNP surface, we employed nanoparticle tracking analysis. Specifically, the lowest particle count was seen in the 0.5 % PEG composition, registering at 0.3x10^11^ particles/mL after transferrin conjugation. This was followed by the 1.5 % Tf-LNPs, which had 1.3x10^11^ particles/mL, and lastly, the formulation containing the highest PEGylated lipid concentration had 4.5x10^11^ particles/mL after Tf-LNP purification. It is important to mention that the NTA measurements for the 0.5 % formulation indicated a high signal-to-noise ratio, which could introduce potential inaccuracies in the measurement for the respective formulation. The measured number of LNPs by NTA exhibited a similar range compared to Ray and coworkers, registering around 1x10^11^ particles/mL using a 3-fold concentration of lipid for synthesis to achieve an Onpattro®-like lipid composition capable of crossing the brain-blood barrier. [16] Corroborating the findings of a study by Li et al., which discussed payload distribution in mRNA LNPs, we observed an increase in LNP size when the concentration of PEG was reduced. This decrease in size can be attributed to a less pronounced shielding effect of PEG before and during the buffer exchange. Consequently, this diminished shielding results in colloidal instability, which in turn causes enhanced particle fusion and fewer shedding events, ultimately leading to an decrease in LNP number and increase in LNP size. [23] Furthermore, it’s possible that, due to the instability and differences in LNP membrane composition, the nanoparticles with low PEG concentration lost integrity or interacted with the filter membrane during purification, consequently reducing the measured particle number. To ascertain the average number of surface-bound transferrin molecules on LNPs, we combined nanoparticle tracking analysis (NTA) with ELISA measurements of transferrin concentrations. Interestingly, the 2.5 % PEGylated formulation revealed a lower number of transferrin molecules per particle—around 31.7—despite having a higher overall transferrin concentration compared with 1.5 % formulation. The 1.5 % Tf-LNPs displayed nearly double the average number of surface-bound transferrin molecules per particle compared with its 2.5 % counterpart. We hypothesize that larger LNPs with strongly increased surface areas, may facilitate greater binding of transferrin molecules. This enhanced binding capacity is anticipated even with a lower percentage of PEGylated lipids and despite a higher number of accessible coupling sites in the 2.5 % PEGylated lipid formulation. Additionally, we consider the possibility of a saturation effect that might restrict the maximal transferrin binding to the LNPs’ surface. This potential saturation may be observable in formulations containing a minimum of 1.5 % PEGylated lipids. Our findings are in line with a theoretical estimation by Yang and colleagues, who calculated an average of 46 transferrin molecules per LNP, thus confirming the validity of our approach. [24] A method for quantifying transferrin molecules on the nanoparticle surface has been previously established by Ishida and colleagues. They utilized a concentration of 0.021 mol % in the context of solid tumor treatment, employing both a phosphorus method and Iod ^125^I labeling of transferrin to measure an average of 26 transferrin molecules per liposome. [25] It is crucial to consider that represented values show an average over millions of particles; in the context of mRNA LNPs, the distribution of PEGylated lipids is not necessarily uniform across all particles. [23] Multiple research groups, including ours, have reported comparable findings. We propose that the reduced presence of transferrin molecules on the surface of LNPs with higher percentages of PEGylated lipid is likely due to transferrin’s inherent properties, namely its high molecular weight of 80 kDa and its negative charge at physiological pH values. The size and charge might lead to steric hindrance and electrostatic repulsion, which could result in a saturation effect on the LNP surface. This could consequently limit the influence of varying PEGylated lipid concentrations on the carrier’s targeting efficacy.

### 3.2 LNP characterization

In this section, we present a comprehensive characterization of LNPs, examining factors such as hydrodynamic diameter, surface charge as indicated by zeta potential, PDI, and encapsulation efficiency. Our aim is to understand the implications of varying PEGylated lipid concentrations in LNP formulations. This evaluation serves as a preliminary step for assessing how well the targeting might work. We envision that GATA-3 silencing LNPs will ultimately be administered to patients locally, either through nebulizers or as a dry powder. However, it’s crucial to consider specific characteristics when aiming for lung-based delivery, such as the size of the particles, their charge, and the extent of their PEGylation. [26,27] In terms of mucus penetration, studies have indicated that particles sized between 100 nm and 200 nm can penetrate through mucus rapidly, while particles smaller than 100 nm demonstrate a low alveolar macrophage uptake of less than 4 %.[10,11] Considering that both the mucus and macrophage surface membranes contain a significant number of negatively charged groups, a nanoparticle with a negative charge is expected to prevent electrostatic attraction. However, excessively negatively charged particles may induce repulsive interactions, adversely affecting mucus penetration and cellular internalization. [28].

**Fig. 1:**
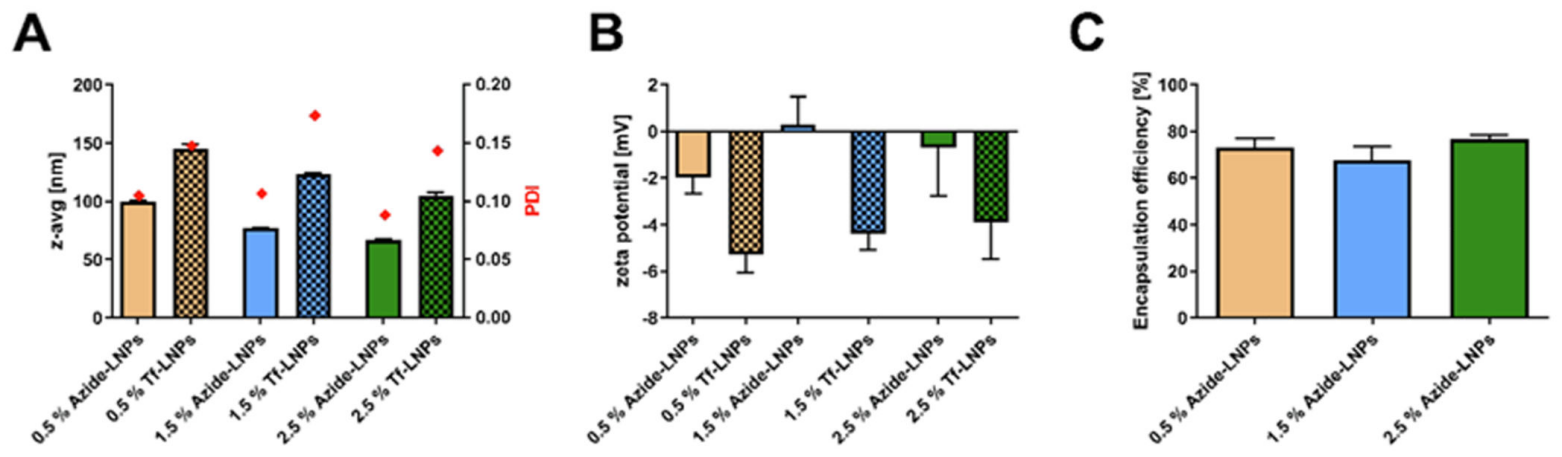
Characterizing LNPs regarding hydrodynamic diameter, polydispersity index (PDI), zeta potential and encapsulation efficiency. LNPs were prepared with 100 pmol GATA3 siRNA at an N/P ratio of 3. The hydrodynamic diameter and PDI **(panel A)** were assessed using DLS at a pH of 7.4. The zeta potential **(panel B)** was determined using PALSat a pH of 7.4. The encapsulation efficiency **(panel C)** was evaluated by diluting the LNPs in TE-buffer followed by a 10-minute incubation with a 1:100 dilution of RiboGreen assay reagent, with readings taken using a plate reader. The standard curve was performed in TE buffer. (Data indicates mean ± SD, n = 3).

In terms of hydrodynamic diameter, we observed a decrease in the hydrodynamic diameter (Fig. 1A) of crude azido-LNPs from ~ 100 nm to ~ 66 nm when increasing the concentration of PEGylated lipids from 0.5 % to 2.5 %. The formation of ~ 77 nm large LNPs was measured when formulating LNP with a 1.5 % PEGylated lipid concentration. These finding are echoed by Kim and colleagues who also observed that the size of LNPs depends on the concentration of PEGylated lipid. They increased the PEGylated lipid concentration from 1.5 % to 4.5 % in an Onpattro®-like LNP formulation and noted a size decrease of about 30 nm. Sarode and coworkers also examined the impact of PEGylation on LNP characteristics and gene silencing capabilities, finding trends similar to those observed in our study. [29,30] The background of this relationship was also investigated by Li and colleagues. They showed that events where mRNA LNPs merge are more likely during buffer exchange and pH increase when the concentration of PEGylated lipid is lower. This leads to the formation of larger LNPs. [23] These larger LNPs, in turn, exhibit less colloidal stability.[31].

Additionally, we evaluated the PDI (Fig. 1A), noting low initial values of 0.1 before transferrin conjugation took place. Subsequent to transferrin conjugation, we observed increases in both size and PDI across all formulations. The size increase was consistently around 50 nm across all formulations we tested. As a result, we measured hydrodynamic diameters of approximately 145 nm, 123 nm, and 108 nm for formulations with PEGylated lipid concentrations of 0.5 %, 1.5 %, and 2.5 %, respectively. Furthermore, there was a negative correlation between the size of the LNPs and their concentration, with the highest particle concentration observed in the 2.5 % PEGylated lipid composition. This could be due to the inherently smaller size of these lipid nanoparticles, particle aggregation, or LNP disassembly occurring during the purification process. To determine if significant cross-linking of the LNPs occurred due to multiple DBCO molecules on the transferrin molecules, we analyzed the DLS size distribution. We observed no additional peaks at larger sizes, as demonstrated in Figure S4. Given that these sizes are within the range of 100 nm to 200 nm, they should be conducive to efficient mucus penetration, and the overall increase can be explained by a layer of Tf on the particle surface. The size increase observed aligns with the findings from low-energy electron holography imaging, which show transferrin glycoproteins itself varying in size from 4 to 12.5 nm depending on their conformation. [32] Furthermore, the PDI increased to approximately 0.15 in all formulations, a value still considered indicative of a low and monodisperse particle distribution. This upward trend in PDI values after transferrin conjugation was also reported by Lin and colleagues, who documented an increase from 0.136 to 0.216. [20] To rule out the possible impact of shear stress—a known factor in aggregate formation—on LNP size, we maintained consistent stirring and centrifugation durations for Tf-LNPs as well as for azide-LNPs. [33] Comparing the size increase with literature values revealed that Yang et al. observed for their studies on Tf-LNP conjugates for breast cancer treatment a particle size increase of approximately 20 nm. These increases occurred when using DSPE-PEG-Maleimide for conjugation with varying LNP lipid compositions. [24].

For zeta potential all crude azide-LNPs displayed negative surface potentials as depicted in Fig. 1B. These surface potentials, which ranged from ~ -2 mV for 0.5 % PEG-azide LNPs to 0 mV for 1.5 % azide-PEG LNPs and –1 mV for 2.5 % PEG-azide LNPs, align with those commonly found in Onpattro®-like MC-3-based formulations. [13] Transferrin conjugation decreased the surface potential by 3–4 mV due to the inherently negative charge of the transferrin protein. This led to a final zeta potential of –4 to –5 mV, suggesting favorable conditions for effective mucus penetration after pulmonary administration *in vivo*. Li et al. also reported a zeta potential decrease by 5 mV, while Yang and colleagues observed a lower reduction, specifically a 1 mV decrease in zeta potential. [20,24].

Studies from our lab involving transferrin-chitosan conjugation have shown comparable trends, including a decrease in zeta potential from 24.1 to 16.1 mV, which varied based on the amount of transferrin used for conjugation. Additionally, we observed size increases from 120 nm to 142 nm and a rise in PDI from 0.194 to 0.251.[34] With regard to encapsulation efficiency, as shown in Fig. 1C, we measured efficiencies between 75 % and 80 %. Commonly, these values are largely influenced by the formulation method used, especially in the context of LNP formulations. [35].

### 3.3 Cellular uptake

The cellular uptake of LNPs is heavily influenced by the formation of a protein corona surrounding the LNPs. This relationship was clearly demonstrated in a study by Dilliard et al., which showed that the protein corona formation, depending on the lipid composition, influenced the distribution of LNPs to different organs. [36] The Onpattro® formulation leverages this phenomenon by encouraging the development of an apolipoprotein (ApoE)-rich corona, which notably enhances hepatocyte uptake via low density lipoprotein receptor (LDL-Receptor) pathway following intravenous administration. [37] The influence of protein corona formation after incubation with bronchoalveolar lavage (BAL) fluid was explored by Subramaniam et al. They observed that the cellular uptake of liposomes within epithelial cells and macrophages varied depending on the surface chemistry and composition. An albumin-rich corona led to rapid endocytosis and exocytosis in macrophages, whereas the uptake within epithelial cells was not influenced by incubation with BAL fluid. [38] We hypothesize that by directly conjugating transferrin to LNPs, we can specifically tailor their uptake for T-cell transfection, thereby avoiding the necessity of protein corona formation for macrophage uptake. Motamedi et al. and our previous work demonstrated that the expression of TfR on the surface of CD4 + Th_2_ cells is enhanced following activation with CD3/CD28 antibodies, a process similar to the activation of T-cells observed in asthma. [39,40].

**Fig. 2:**
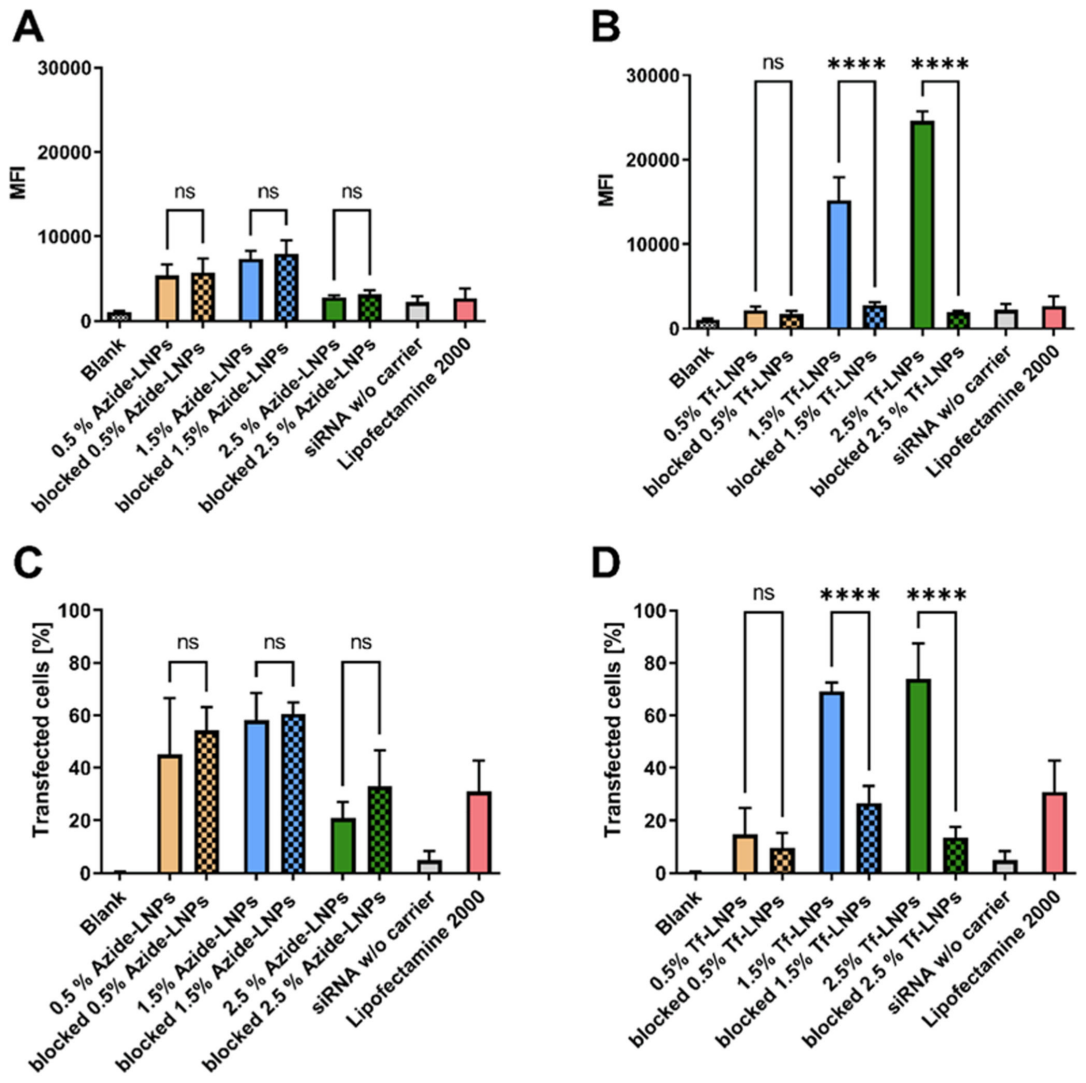
Cellular uptake in Jurkat cells after siRNA LNP treatment. Jurkat cells were incubated with LNPs loaded with 100 pmol of AF647-labeled siRNA at a N/P ratio of 3, as described above. 24 h after-transfection, the median fluorescence intensity (MFI) **(panels A and B)** and the proportion of transfected cells **(panels C and D)** were assessed using flow cytometry for both azide-LNPs and Tf-LNPs. Untreated cells are denoted as “Blank”, serving as the negative control, while cells transfected with 100 pmol of AF647-labeled siRNA using Lipofectamine 2000 served as the positive control. (Data points indicate mean ± SD, n = 3; One-way ANOVA, **, p < 0.01, ***, p < 0.001, ****, p < 0.0001).

**Fig. 3:**
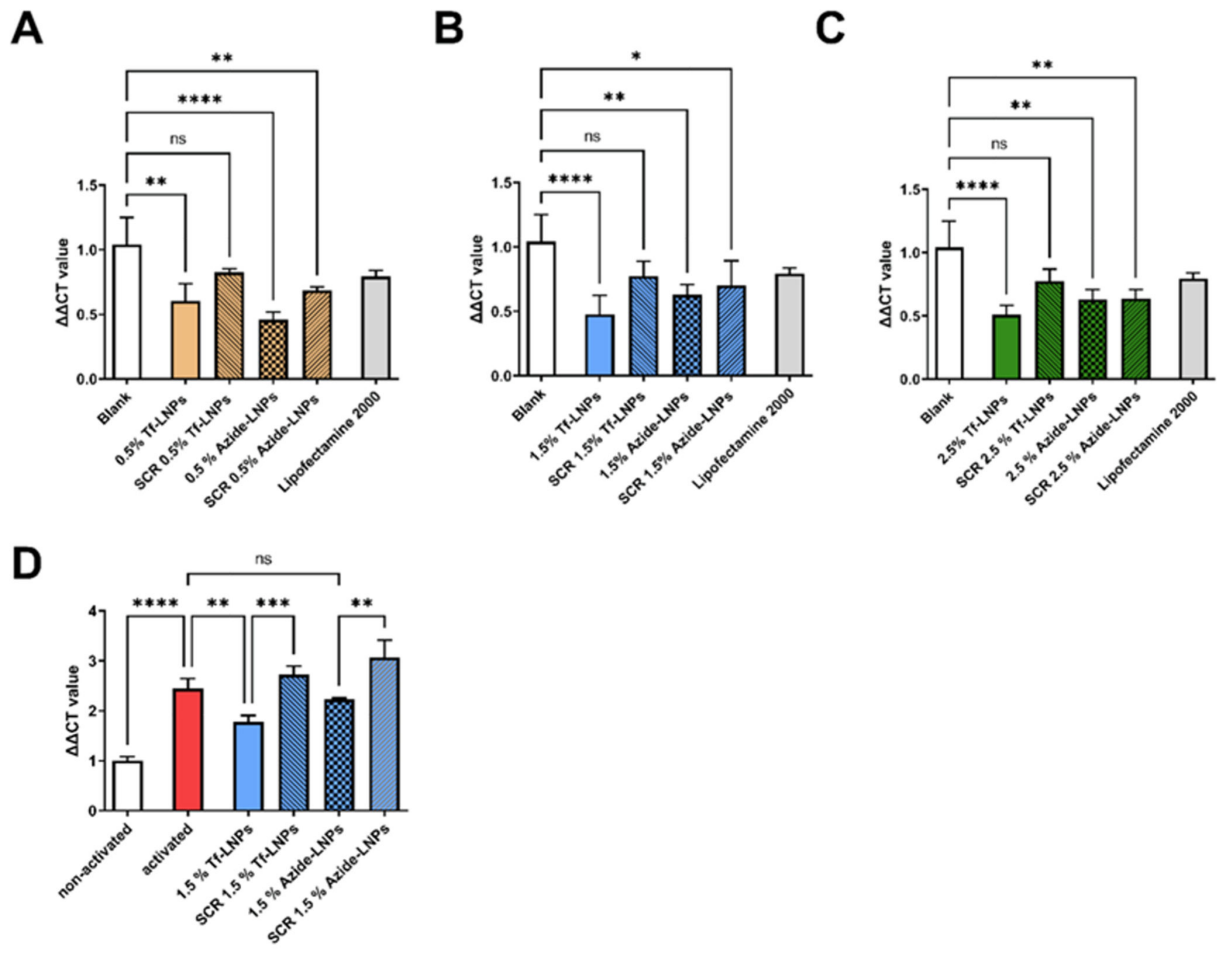
GATA3 knockdown in Jurkat (**panels A, B, C with increasing Tf modification**) and primary CD4 + Th_2_ (**panel D**) cells treated with 100 pmol GATA3 siRNA containing LNPs at N/P 3. GATA3 expression was evaluated by qRT-PCR and normalized against *β-actin* expression. Blank represents non-treated cells. Negative control (NC) cells were transfected with respective LNPs containing scrambled siRNA (Data points indicate mean ± SD, n = 3, N = 3 One-way ANOVA, Tukey posthoc Test *, p < 0.05,**, p < 0.01, ***, p < 0.001, ****, p < 0.0001).

To enhance cellular uptake into Th_2_-cells, we hijacked the TfR pathway. The TfR is well-known to undergo clathrin-dependent endocytosis upon binding with transferrin, facilitating iron uptake into cells physiologically. Subsequent to internalization, TfR is typically recycled back to the cell surface avoiding trafficking to the late endosome or lysosome. [41] Given that LNPs are recognized to escape from endosomes before and after reaching the late stages of endosomal maturation, this pathway proves advantageous for active targeting. [42–44] Alternatively, it’s possible that strong binding of the LNPs to the TfR results in their release back to the membrane. Furthermore, to decrease unspecific uptake and facilitate the active targeting by decreasing the protein corona formation, we employed PEGylated lipids for conjugation. [18] We performed cellular uptake studies using Jurkat cells. The T-lymphocyte cell line is often used in studies focused on nanocarrier targeting via the TfR pathway and known to express TfR in a large number. [8,34,45] These cells were transfected with LNPs formulated with a blend of fluorescently labeled siRNA and scrambled siRNA. As shown in Fig. 2A, in the absence of an active targeting ligand, the cellular uptake of LNPs varies depending on the concentration of PEGylated lipids in the formulations. The highest cellular uptake was measured after transfection with 1.5 % azide-LNPs. However, an increment in the percentage of PEGylated lipids resulted in decreased cellular uptake, a phenomenon attributed to so-called “*PEG dilemma*”: the stealth effect hinders cellular uptake and endosomal escape, albeit enhancing colloidal stability and increasing blood circulation. [46,47] The formulation with 0.5 % PEGylated lipid exhibited reduced uptake, which might be attributed to colloidal instability of the formulation. Conversely, the inclusion of a targeting ligand amplified cellular uptake significantly. This trend was again dependent on the PEGylated lipid concentration utilized. Highest uptake was achieved when transfecting cells with a lipid composition of 2.5 % PEGylated lipid. Given that the number of transferrin molecules present on the surface of LNPs remained higher for 1.5 % compared to 2.5 % formulations, we postulate that the increased cellular uptake of LNPs is attributed to the number of LNPs and siRNA internalized. Interestingly, the 0.5 % PEGylated lipid formulation did not offer any apparent advantages. We visualized cellular uptake using a 1.5 % PEGylated lipid formulation. As depicted in Figure S5, Tf-LNPs were extensively internalized after 24 h, in contrast to only minimal signals observed in formulations lacking the transferrin ligand. Given that siRNA-containing LNPs are known to escape from endosomes at a limited rate (1–2 %), significant colocalization with endosomes was not surprising. [44] To conclude whether the enhanced uptake was due to the targeting ligand or altered physicochemical properties, we conducted a competition assay. This involved adding an excess of the targeting ligand, transferrin, 2 h prior to transfection. As shown in Fig. 2A and 2B, the addition of excess transferrin had no impact on azide-LNP uptake but led to a significant reduction in the uptake of Tf-LNPs, even falling below the levels observed with unmodified LNPs. While we cannot conclusively predict the impact of DBCO incorporation on transferrin conformation, the preserved functional efficacy observed suggests that any conformational changes are not substantially harmful. Lipofectamine demonstrated overall low cellular uptake in T-cells, which are known to be difficult to transfect, as shown by ourselves [48] and others before.[49]. Fig. 3.

Interestingly, the percentage of transfected cells, shown in Fig. 2C and 2D, did not correlate with the measured fluorescence intensity values. For the 0.5 % and 1.5 % formulations, azide-LNPs achieved a transfection rate of over 50 %, contrasting with the 20–60 % transfection rate observed for Tf-LNPs. The discrepancy may be due to larger number of particles being internalized by cells when using Tf-LNPs. Significantly, the number of cells transfected by Tf-LNPs also showed a notable decrease following the competition assay. This increase in siRNA transfection efficiency is consistent with studies that have compared the uptake of labeled Tf-PEI to its unlabeled version in Jurkat cells. [8] In line with our results, research on MV4-11, Kasumi-1 and OCl-AML3 cells also indicated some degree of non-specific uptake happening after a 4-hour period. [50] However, given that mean fluorescence intensity (MFI) was not assessed, drawing direct comparisons proves challenging. Another factor impacting cellular uptake and endosomal escape is the ‘shedding effect’ of PEGylated lipids in LNPs. These lipids are known to diffuse more rapidly from the nanoparticle, depending on factors such as C-chain length *in vivo*. Given the importance of this effect, we opted for DSPE-PEG with an 18-carbon chain length over PEG-DMG, which has a 14-carbon chain length and is used in the Onpattro® formulation. Studies have shown that the half-life (t ½) of DMG-PEG-based formulations was around 1.33 h, while the longest recorded half-life exceeded 24 h using DSPE-PEG. In our specific context, the rapid diffusion of PEGylated lipids from the LNP might negatively impact its targeting ability. [51].

### 3.4 GATA3 knockdown

To assess the knockdown efficiency of our targeted LNP formulations, we employed Jurkat cells (Fig. 3A-C), as they are known to overexpress GATA3 homeostatically. [52].

Following transfection with the 1.5 % and 2.5 % Tf-LNP formulations (Fig. 3B, C), a notable reduction in GATA3 mRNA levels was observed, with 1.5 % Tf-LNPs achieving a knockdown efficiency exceeding 50 %. A modest decrease was also noted in samples transfected with scrambled siRNA, suggesting that either the scrambled siRNA or the LNP itself may have some influence on GATA3 expression. Further studies are necessary to investigate this phenomenon. Considering that Lipofectamine already exhibited low cellular uptake, the observed insufficient knockdown is not surprising. Interestingly, the 0.5 % Tf-LNP formulation lagged in performance compared to its azide-LNP counterpart (Fig. 3A). Given that no increase in cellular uptake was observed following transferrin conjugation, we hypothesize that the reduction in GATA3 mRNA levels—approximately 35 – 40 % GATA3 knockdown—could be attributed to non-transferrin mediated uptake and superior endosomal escape capabilities at lower PEG concentrations. For the 2.5 % Tf-LNP formulation, a decrease in knockdown efficacy was observed, which we believe is due to decreased endosomal escape abilities associated with higher PEG concentrations. This aligns with existing literature: for instance, Kim et al. reported a greater than 50 % decline in efficiency when increasing the PEGylated lipid concentration from 1.5 % to 2.5 % in mRNA LNPs. [17] Similarly, a high-throughput study found that LNPs with 3 and 5 mol % DSPE-PEG concentrations exhibited a 50 % reduction in knockdown efficiency [18]. Our observations regarding the comparable transfection efficiencies between targeted and non-targeted LNPs are consistent with findings from Kheirolomoom et al., who reported increased mRNA transfection efficiency in Jurkat cells treated with anti-CD3-conjugated lipid nanoparticles. [14] Therefore, our results suggest that while transferrin conjugation enhances specificity, the overall knockdown efficacy may be influenced by other factors, including PEGylation levels and non-specific cellular uptake mechanisms.

To evaluate the effectiveness of transferrin-conjugated lipid nanoparticles (Tf-LNPs) in silencing GATA3 compared to their unconjugated counterparts within a more relevant patient setting, we conducted an *ex vivo* study in CD4^+^ activated Th_2_ cells isolated from PBMCs. Drawing on the insights from experiments with Jurkat cells, we utilized a 1.5 % PEG lipid composition as the optimal formulation. As depicted in Figure 3D, a GATA3 knockdown of 50 % compared to non-activated Th_2_ cells was achieved, mirroring the results observed in Jurkat cells. Conversely, the azide-LNP formulation failed to induce a substantial reduction in GATA3 expression compared to the activated Th_2_ cells. Formulations employing non-coding RNA resulted in an increase in GATA3 expression. Further investigation is required to determine whether this effect is attributable to the RNA or the lipid composition. Comparatively, our LNP formulations outperformed polymeric delivery systems such as Tf-PEI in terms of knockdown efficiency, while maintaining a safety profile supported by clinical approval of LNPs. [10] Additionally, we achieved a GATA3 mRNA reduction surpassing the levels reported for DNAzyme technology, which has successfully completed clinical phase 2b trials for the treatment of type 2 asthmatic disease. [53].

## 4 Conclusion

In conclusion, we have provided insights into the targeting capabilities of LNPs after modification, specifically measuring the average number of transferrin molecules bound to individual LNPs and the relationship between PEG concentration and the number of bound transferrin molecules. We utilized strain-promoted azide-alkyne cycloaddition for transferrin conjugation and successfully achieved particle characteristics suitable for pulmonary delivery. Additionally, we were able to demonstrate cellular uptake in T cells dependent on the targeting ligand concentration and achieved > 50 % knockdown of the therapeutically relevant GATA3 gene *in vitro* and *ex vivo*.

The saturation effect we noted suggests potential limitations in modifying LNP surfaces, particularly when using PEGylated lipids as spacers for active targeting. These findings lay the groundwork for future studies, investigating Tf-LNP efficiency in T cell targeting and for the treatment of inflammatory diseases in *in vivo* models.

## Supplementary Material

Supplementary information

## Figures and Tables

**Table 1:** Transferrin conjugation was evaluated by NTA and transferrin ELISA. The particle concentration of siRNA loaded LNPs containing 100 pmol GATA3 siRNA at an N/P ratio of 3 was measured by NTA. The transferrin concentration was evaluated via transferrin ELISA. The average of transferrin per LNP as well as coupling efficiency was measured as described above. (Data points indicate mean ± SD, n = 3, * = below detection limit).

Sample	LNP concentration [10^11 particles/ml]	Mean Transferrin mass concentration [µg/mL]	Average Transferrin molecules per LNP
**0.5 % Tf-LNPs**	0.26 ± 0.2	1.16	*
**1.5 % Tf-LNPs**	1.3 ± 0.7	1.88	65.0 ± 6.5
**2.5 % Tf-LNPs**	4.5 ± 0.6	7.30	31.7 ± 4.5

## Data Availability

Data will be made available on request.
