# Supplementary material for: Tailoring lipid nanoparticles for T-cell targeting in allergic asthma: Insights into efficacy and specificity
Source: Eur J Pharm Biopharm. Author manuscript; Available in PMC 2024 Oct 24. (PMC7616735; doi:10.1016/j.ejpb.2024.114242)
Supplement: Supplementary information [file EMS199500-supplement-Supplementary_information.docx]

**Supplementary section**

**Table S1** Lipid compositions used in this study

| **Sample** | **D-Lin-MC3-DMA [%]** | **DSPC [%]** | **Cholesterol [%]** | **DSPE-PEG(2000)-Azide [%]** |
| --- | --- | --- | --- | --- |
|  |  |  |  |  |
| **0.5% Tf-LNPs** | 50 | 10 | 39.5 | 0.5 |
| **1.5% Tf-LNPs** | 50 | 10 | 38.5 | 1.5 |
| **2.5% Tf-LNPs** | 50 | 10 | 37.5 | 2.5 |


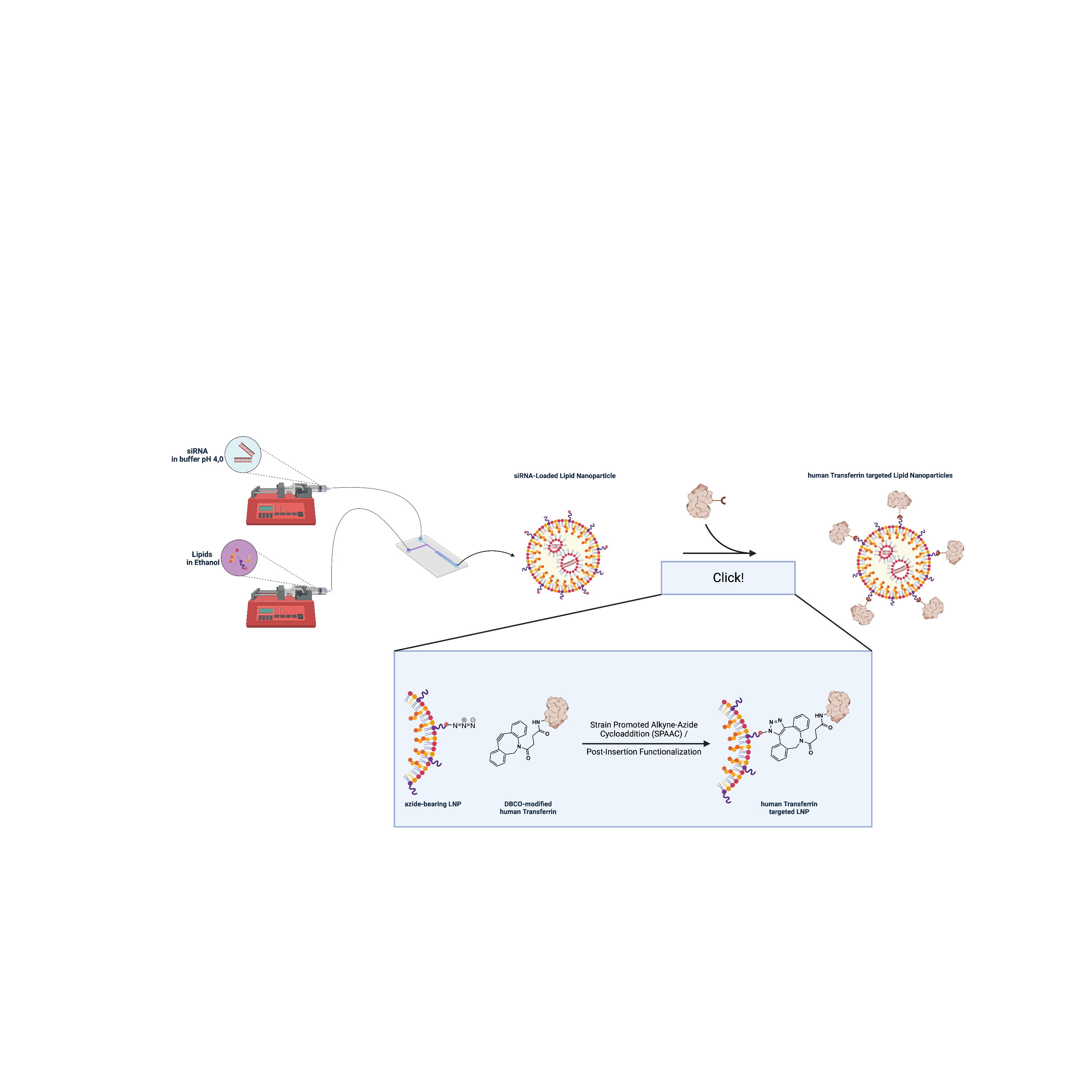


**Figure S1** Schematic illustration of SPAAC coupling reaction

**Table S2** Tf-DBCO purity. DBCO absorption in the sample and flow through measured at 309 nm.

|  | Absorption at λ = 309 nm |
| --- | --- |
| Third flow through | 0.03 |
| Tf-DBCO solution | 14.35 |

**Figure S3** Tf-LNP purity. Free Tf-DBCO in the flow through of modified LNPs after several washing steps quantified by ELISA (Data points indicate mean ± SD)


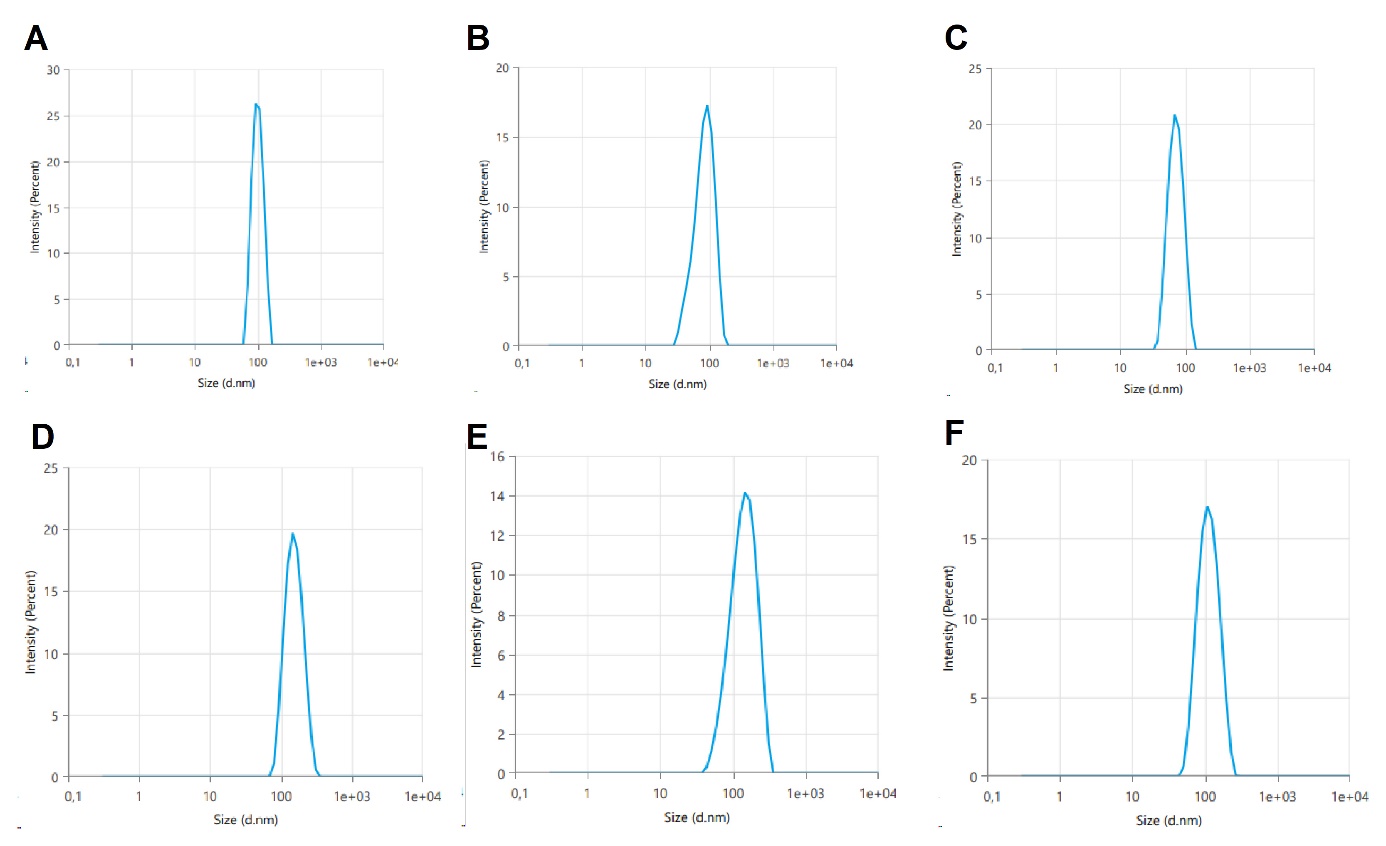


**Figure S4** DLS measurements of LNPs. Measurements were shown for-LNPs formulated with 0.5 % **(Panel A),** 1.5 % **(Panel B)**, and 2.5 % **(Panel C)** PEGylated lipid as well as for Tf-LNPs formulated with 0.5 % **(Panel D),** 1.5 % **(Panel E)**, and 2.5 % **(Panel F)** PEGylated lipid.


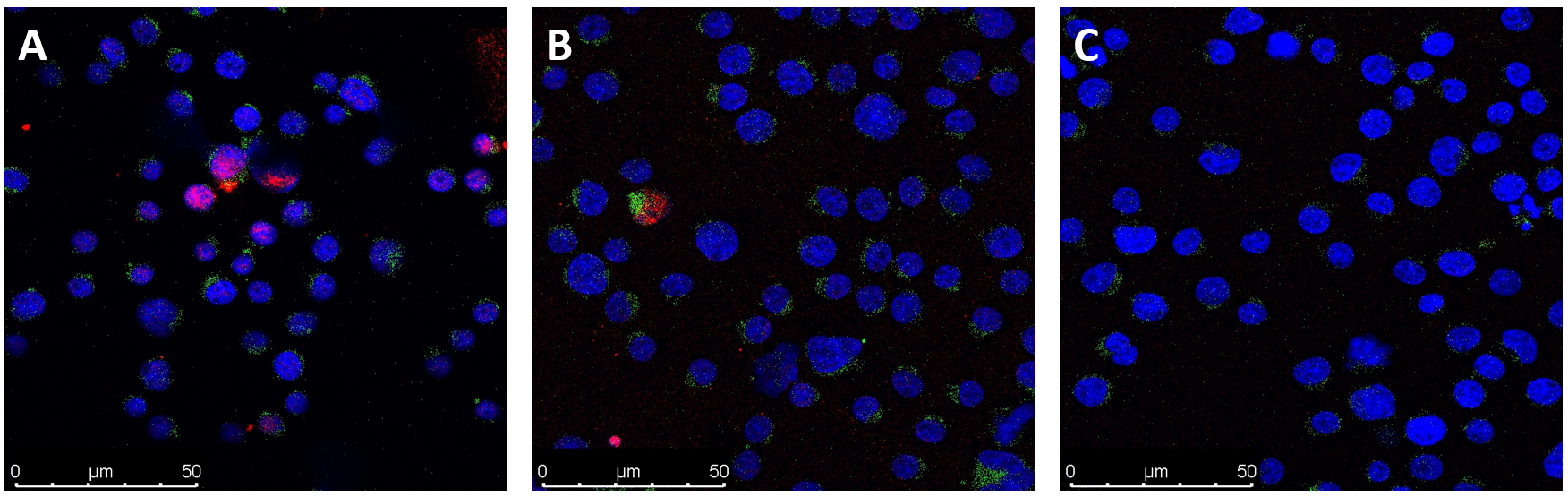


**Figure S5** Cellular distribution of LNPs in Jurkat cells visualized by confocal microscopy. Confocal microscopy was applied 24 hours after treatment with Azide-LNPs (**Panel A**) and Tf-LNPs (**Panel B**). AF647-labeled siRNA is shown in red, Lysotracker Yellow in green, and DAPI-stained nuclei in blue. Untreated cells were evaluated as a control (**Panel C**).


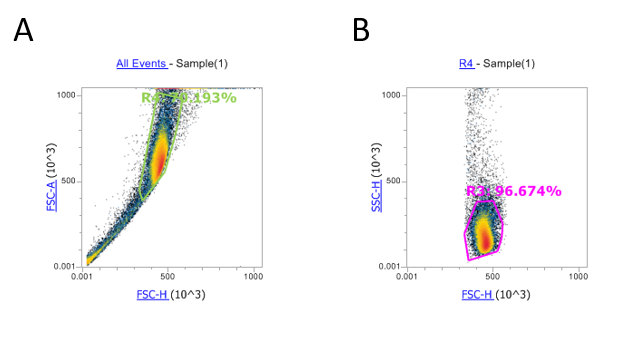


**Figure S6** Gating strategy for cellular uptake experiments (panel A and B)


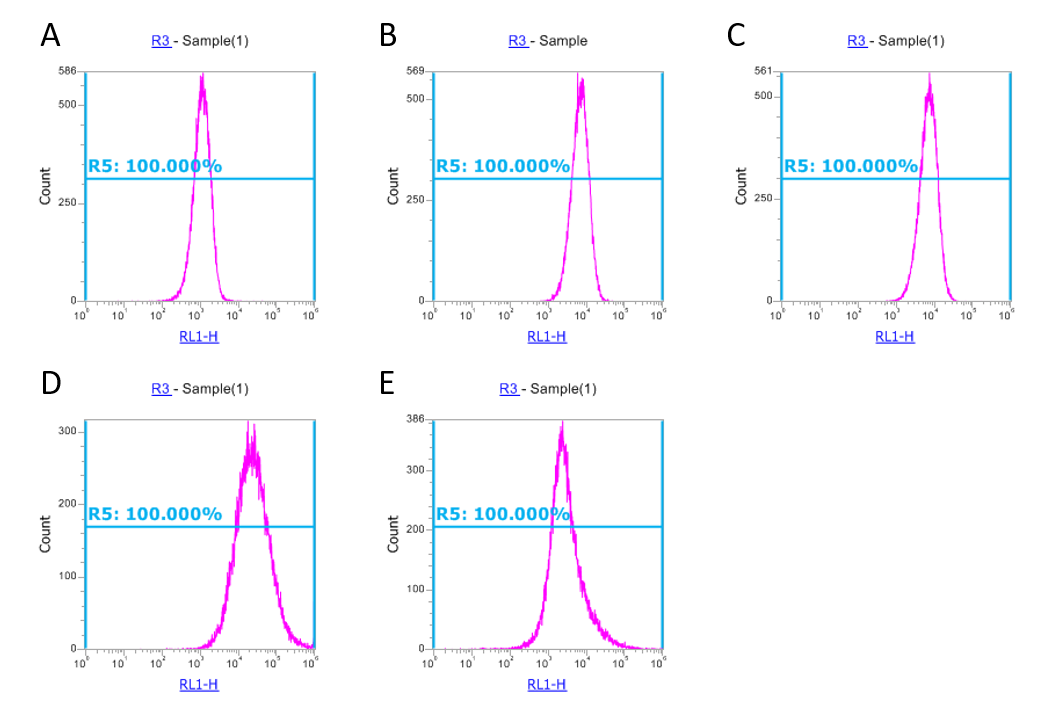


**Figure S7** Histogram plots of cellular uptake in Jurkat Cells 24 h after siRNA LNP treatment shown exemplarily for 1.5 % PEGylated lipid concentration. Jurkat cells were incubated with LNPs loaded with 100 pmol of AF647-labeled siRNA at a N/P ratio of 3 for Azide-LNPs (**panel B)** and Tf-LNPs **(panel D).** Blank represents untreated cells (**panels A).** TfR competition was performed **(panel C and E)** for each LNP formulation respectively.
